# Promotion effect of TGF-β-Zfp423-ApoD pathway on lip sensory recovery after nerve sacrifice caused by nerve collateral compensation

**DOI:** 10.1038/s41368-023-00230-7

**Published:** 2023-06-08

**Authors:** Pingchuan Ma, Gaowei Zhang, Su Chen, Cheng Miao, Yubin Cao, Meng Wang, Wenwen Liu, Jiefei Shen, Patrick Ming-Kuen Tang, Yi Men, Li Ye, Chunjie Li

**Affiliations:** 1 State Key Laboratory of Oral Diseases & National Clinical Research Center for Oral Diseases & Department of Head and Neck Oncology, West China Hospital of Stomatology, Sichuan University, Chengdu, China; 2 State Key Laboratory of Oral Diseases & National Clinical Research Center for Oral Diseases & Department of Oral and Maxillofacial Surgery, West China Hospital of Stomatology, Sichuan University, Chengdu, China; 3 Department of Medical Record, West China Hospital of Stomatology, Sichuan University, Chengdu, China; 4 Department of Geriatric Dentistry, Peking University School and Hospital of Stomatology, Beijing, China; 5 State Key Laboratory of Oral Diseases & National Clinical Research Center for Oral Diseases & Department of Prosthodontics, West China Hospital of Stomatology, Sichuan University, Chengdu, China; 6 Department of Medicine & Therapeutics, Li Ka Shing Institute of Health Sciences, and Lui Che Woo Institute of Innovative Medicine & Department of Anatomical and Cellular Pathology, State Key Laboratory of Translational Oncology, Prince of Wales Hospital, The Chinese University of Hong Kong, Shatin, Hong Kong China

**Keywords:** Chronic pain, Biophysical chemistry

## Abstract

Resection of oral and maxillofacial tumors is often accompanied by the inferior alveolar nerve neurectomy, resulting in abnormal sensation in lower lip. It is generally believed that spontaneous sensory recovery in this nerve injury is difficult. However, during our follow-up, patients with inferior alveolar nerve sacrifice showed different degrees of lower lip sensory recovery. In this study, a prospective cohort study was conducted to demonstrate this phenomenon and analyze the factors influencing sensory recovery. A mental nerve transection model of Thy1-YFP mice and tissue clearing technique were used to explore possible mechanisms in this process. Gene silencing and overexpression experiments were then conducted to detect the changes in cell morphology and molecular markers. In our follow-up, 75% of patients with unilateral inferior alveolar nerve neurectomy had complete sensory recovery of the lower lip 12 months postoperatively. Patients with younger age, malignant tumors, and preservation of ipsilateral buccal and lingual nerves had a shorter recovery time. The buccal nerve collateral sprouting compensation was observed in the lower lip tissue of Thy1-YFP mice. ApoD was demonstrated to be involved in axon growth and peripheral nerve sensory recovery in the animal model. TGF-β inhibited the expression of STAT3 and the transcription of ApoD in Schwann cells through Zfp423. Overall, after sacrificing the inferior alveolar nerve, the collateral compensation of the ipsilateral buccal nerve could innervate the sensation. And this process was regulated by TGF-β-Zfp423-ApoD pathway.

## Introduction

The inferior alveolar nerve (IAN) is a branch of the mandibular division of the trigeminal nerve (V3), and its terminal branch, the mental nerve (MN), emerges from the mental foramen to distribute to the skin and mucosa of the lower lip.^1^ As IAN passes through the mandibular canal in the medullary part of the mandible, mandibulectomy, no matter marginal or segmental, is likely to cause a segmental defect of the IAN, leading to sensory dysfunction in the innervated area and a decline in patients’ quality of life.^2^ Although previous studies have indicated that the peripheral nervous system (PNS) has a potential for regeneration after injuries, spontaneous sensory recovery is believed to be challenging and difficult.^3,4^ A possible reason is that the long distance between the target tissue and nerve breakpoint makes it hard to re-establish a connection.^5^ Although autologous nerve grafting is currently the gold standard for reconstructing segmental peripheral nerve defects,^6^ the issues of donor site morbidity and limited nerve supply could not be neglected and the recovery results were not satisfactory.^7^ However, during clinical follow-up, we found that some patients undergoing IAN sacrifice exhibited different degrees of lower lip sensory recovery.

To further explore the changes in peripheral nerves during the sensory recovery of the lower lip, we adopted tissue-clearing technology. Tissue-clearing techniques remain an option for decolorizing pigment elements and eliminating refractive index (RI) mismatch within the different components of tissues to achieve transparency.^8^ Without obstruction by irrelevant hard and soft tissues, targeted tissue variation could be observed through this pattern using fluorescence preservation.^9^ The images of the peripheral nervous system (PNS) and central nervous system (CNS) could be identified in three-dimensional views, especially with the aid of fluorescence.^10^ Combined with Thy1-YFP-M (with yellow fluorescence), the specific nerve change can be reflected for observation.

Schwann cells (SCs), myelinated glial cells of the PNS, play a vital role in promoting nerve repair and functional recovery by converting to denervated SCs in Wallerian degeneration.^11^ Subsequently, repair SCs would clear myelin debris by autophagy, contribute to macrophage-mediated myelin removal during Wallerian degeneration, and aid in axon outgrowth.^12^ Oligodendrocytes and Schwann cells are the most important expression sites of ApoD in the nervous system.^13^ ApoD overexpression has been detected in a variety of central nervous system diseases, such as Alzheimer’s disease, Parkinson’s disease, and Niemann Pick’s disease.^14^ ApoD can inhibit arachidonic acid and phospholipase A2 after nerve stress or injury, limiting the scale and time of inflammation.^15,16^ In addition, ApoD has an antioxidant effect and participates in myelin maintenance and clearance.^17,18^ After peripheral nerve injury (PNI), TGF-β is widely expressed in the early stage and is usually involved in the anti-inflammatory effect after injury.^19^ It also participates in the phenotypic changes of SCs, regulates immune cells, and plays a neuroprotective role.^20^ However, the relationship between TGF-β and ApoD is unexplored.

In this study, we conducted a prospective cohort study aimed to demonstrate spontaneous sensory recovery after IAN sacrifice and explore the possible factors of lower lip sensory recovery through statistical methods. In addition, the potential mechanism of sensory recovery of the lower lip was explored through animal experiments combined with tissue clearing technology.

## Results

### Factors influencing complete lower lip sensory recovery time

A total of 131 patients were enrolled, 23 of whom were excluded for loss of follow-up, cancer recurrence, or improper information. Therefore, 108 patients were included, all with IAN sacrifice (Fig. S1, Table S2). Eighty-one of the included patients exhibited complete sensory recovery of the lower lip at month 12. Statistical analysis showed that patients with younger age, preservation of the ipsilateral buccal or lingual nerve, no radiotherapy, and no chemotherapy had higher proportions of complete lower lip sensory recovery.

**Fig. 1:**
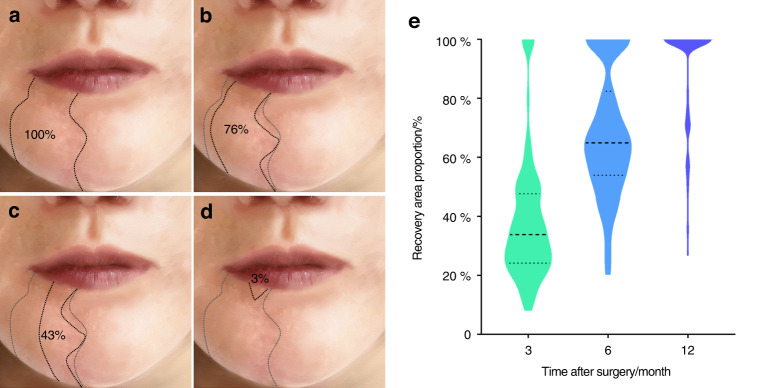
The sensation of lower lip gradually recovered after inferior alveolar nerve sacrifice. **a** Sensory disturbance area diagram of lower lip 1 month after operation. **b** Sensory disturbance area diagram of lower lip 3 months after operation. **c** Sensory disturbance area diagram of lower lip 6 months after operation. **d** Sensory disturbance area diagram of lower lip 1 year after operation. **e** Proportion of lower lip sensory recovery area at 3, 6, and 12 months after surgery based on the area with lower lip sensory disturbance at 1 month after surgery

The proportion of complete lower lip sensory recovery increased over time. Seven (6.5%) and 26 (24.1%) patients fully recovered within three and six months after surgery, respectively. After 12 months, 81 (75%) patients exhibited complete recovery. Figure 1a-d displays the patients’ representative recovery process. In addition, the proportion of lower lip sensation recovery area increased over time (Fig. 1e).

Potential factors affecting sensory recovery time were evaluated. According to univariate analysis, patients with younger age (*P* < 0.001) and conservation of the ipsilateral buccal nerve (BN) (lg HR = −0.335, *P* = 0.003) exhibited a significantly shorter recovery time (Table 1). In addition, multivariate Cox regression indicated that the time of complete lower lip sensory recovery was significantly associated with patients’ age (*P* < 0.001), pathological type (*P* = 0.003), ipsilateral BN removal (*P* = 0.039), and ipsilateral lingual nerve removal (*P* = 0.034) (Table 1).

**Table 1:**
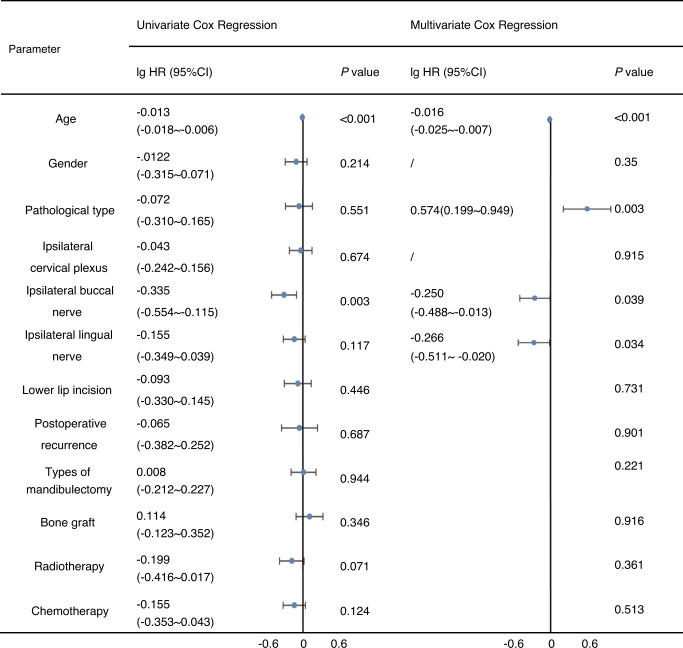
Factors influencing complete lower lip sensory recovery time

*CI* Confidence interval; *HR* Hazard ratio

Figure 1e presents the proportion of patients and their sensory recovery area at different time intervals. Relevant clinical factors for the diminishment of the numbness proportion were evaluated. The results showed that age (*P* < 0.001) could significantly affect sensory recovery proportion three months after IAN sacrifice. Older age (*P* < 0.001) and removal of the ipsilateral buccal nerve (*P* = 0.013) contributed to a significantly smaller proportion of the recovery area six months after surgery (Table S3).

### Reinnervation occurred in the lower lip of rats after mental nerve transection

**Fig. 2:**
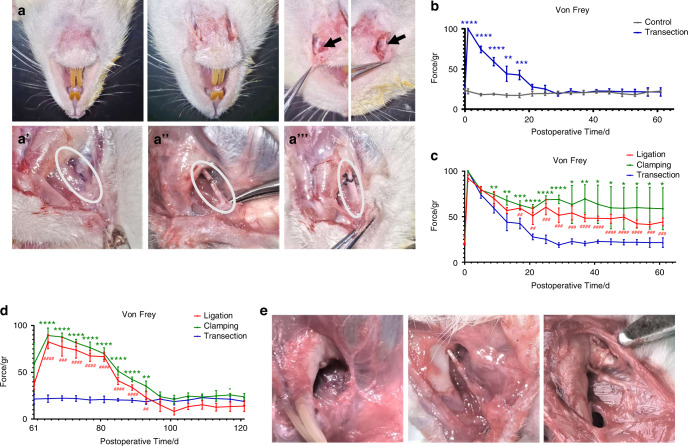
Reinnervation occur in the lower lip of rats after mental nerve injury. **a** Establishment of 3 rat models of mental nerve injury. a’ Transection of right mental nerve; a” Clamping of right mental nerve; a”’ Ligation of right mental nerve. Black arrows indicated exposed bilateral mental nerves. White circles indicated the injured mental nerves. **b** QST analysis showed bilateral lower lip sensation after right mental nerve transection in rats, measured every four days until 2 months after operation (*n* = 5). **c** QST analysis showed right lower lip sensation after 3 kinds of mental nerve injury in rats, measured every four days until 2 months after operation (*n* = 5). **d** QST analysis showed right lower lip sensation of rats in the clamping and ligation group after the right mental nerve transection 2 months after mental nerve injury, measured every four days (*n* = 5). **e** Anatomy of the right mental nerve 2 months after operation in 3 models of mental nerve injury. Note: *Clamping vs Transection; *P < 0.05; **P < 0.01; ***P < 0.001; ****P < 0.0001. #Ligation vs Transection; ^#^*P* < 0.05; ^##^*P* < 0.01; ^###^*P* < 0.001; ^####^*P* < 0.000 1

To further study the mechanism of sensory recovery of the lower lip after IAN transection, we established a rat model of right MN transection (Fig. 2a). A quantitative sensory test (QST) was performed on bilateral lower lips every four days after the operation. The QST results of the lower lips showed that the lower lip sensation gradually recovered over time after the right MN transection, with no significant difference between the bilateral lower lip sensation on the 21st day after the operation (Fig. 2b). QSTs were performed on the right lower lip of rats with three kinds of MN injury: transection, ligation, or clamping. The results showed that the sensory recovery of the lower lip in the transection group was more satisfactory (Fig. 2c). After two months, the right MN in the ligation and clamp groups underwent transection again, and the sensation of the right lower lip was further recovered (Fig. 2d). Meanwhile, we found that, two months after the transection of the right MN in rats, there was some regeneration of the proximal cranial end of the right MN; however, it was not continuous with the lower lip tissue, indicating that the sensory recovery of the lower lip did not depend on the regeneration of the severed MN (Fig. 2e). The above results showed that the sensory recovery of the lower lip was not caused by MN regeneration.

### The buccal nerve and mental nerve innervating the sensory recovery area of the lower lip

**Fig. 3:**
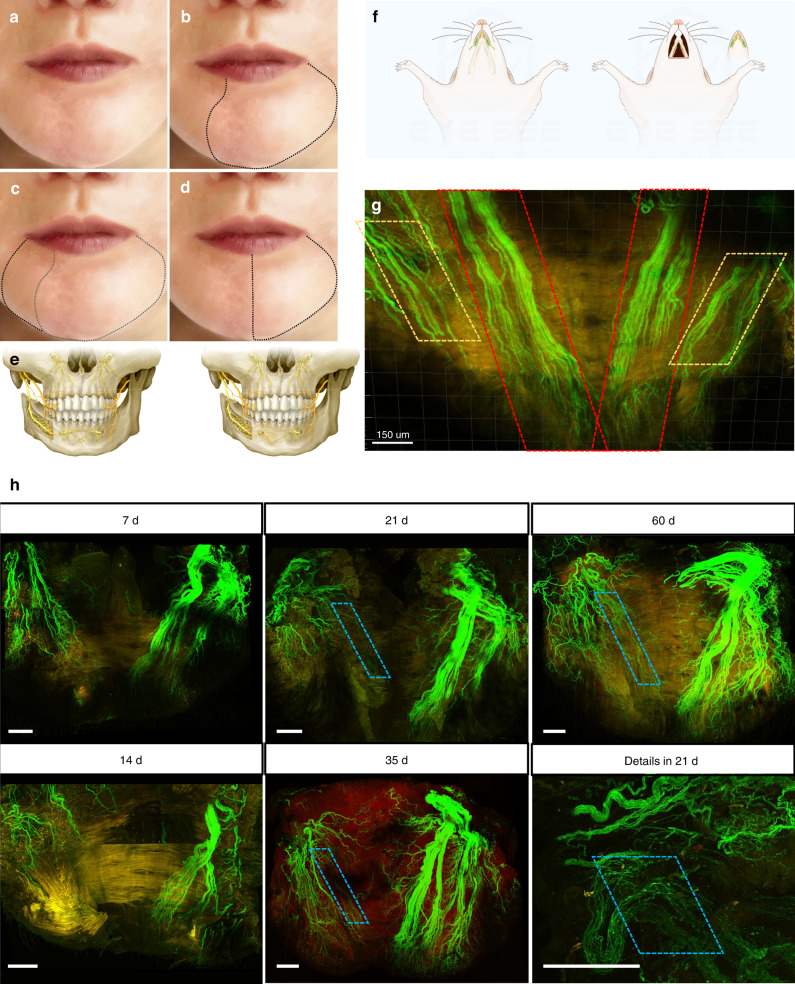
The buccal nerve participates in the sensory recovery of the lower lip through nerve collateral compensation. **a** Sensory disturbance area diagram of the lower lip 12 months after right IAN sacrifice. **b** Sensory disturbance area diagram after left MN block. **c** Sensory disturbance area diagram after left MN and right BN block. **d** Sensory disturbance area diagram after left MN block in normal person. **e** Schematic diagram of lower lip sensation under compensatory innervation of buccal nerve. **f** Schematic diagram of mice lower lip dissection. The buccal nerve was marked with green, and the mental nerve was marked with yellow. **g** Fluorescence display of BNs and MNs after tissue clearing procedure of Thy1-YFP mice lower lip. Bilateral MNs were marked with red boxes and the fibers of BNs were marked with yellow boxes (scale bar: 150 μm). **h** Fluorescence display of BNs and MNs of Thy1-YFP mice lower lip after right MN sacrifice. The collateral compensation was marked with blue boxes (scale bar: 200 μm). The autofluorescence of collateral compensation fibers was inferior to natural intact nerve axon

Ipsilateral BN preservation is an independent factor for the recovery of lower lip sensation, while bilateral MNs can dominate the contralateral sensation across the middle line of the lower lip. To clarify the roles of the BN and MN in the sensory recovery of the lower lip, we implemented nerve blocks in 18 volunteer patients with complete sensory recovery 12 months after IAN transection to detect the possible sensory recovery mechanism. The first eight participants had an ipsilateral MN block, with little (<10%) or no anesthetic area in the ipsilateral lower lip. Considering the limited effects of the ipsilateral MN block, we stopped this procedure in the following observations. Eighteen participants underwent the contralateral MN or IAN block, and we found that part of the ipsilateral lower lip innervated by the resected IAN was numb. These participants also had an ipsilateral BN block, and the numb area extended to the rest of the ipsilateral lower lip (Fig. 3a–d), indicating that ipsilateral BN was vital in the innervation of the sensory function of the lower lip after the alveolar nerve or MN transection, and the contralateral MN may also innervate the midline of the lower lip (Fig. 3e).

To determine the compensatory mechanisms of the trigeminal nerve, we established the right MN transection model of Thy1-YFP mice. The sensory disorder of the lower lip induced by segmental defects measuring >4 mm of MN cannot recover from reinnervation or regeneration of the original severed nerve. In addition, soft tissue samples, including the whole lower lip and chin skin, were dissected and processed by a tissue clearing procedure for nerve compensation mechanism analysis (Fig. 3f). We first characterized significantly advanced Wallerian degeneration on day 7 after MN injury. Samples with an immersed procedure in the medium were imaged by a multiphoton confocal microscope (Leica). We analyzed the nerve structure of the MN trunk and the branch in the lower lip, respectively. The MN (red box) and BN (yellow box) of the lower lip exhibited apparent fluorescence (Fig. 3g). After the MN injury on the right side, fluorescence analysis showed an axonal signal of the MN trunk loss on the injury side. In contrast, intact nerve structures were visualized in BN on the same side and uninjured MN on the control side (Fig. 3h). At the level of the lower lip, two regions were identified by median line: a dark area with no autofluorescence for the Wallerian degeneration of MN distal stump on the injury side and an intact area presenting a natural MN innervation on the control side. To assess the presence of nerve compensation, we analyzed the dark area since all axons degenerated in this region. Twenty-one days after MN injury, new autofluorescence appeared in the dark area (as the blue box indicated). The three-dimensional reconstruction showed that the fluorescence intensity of the emerging nerve was inferior to the undamaged nerve. Maximum radius analysis revealed that axon collaterals from ipsilateral BN, but not original MN, contributed to the nerve compensation. Thirty-five days after injury, the autofluorescence exhibited stronger and clearer nerve structures in the dark area (as the blue box indicates). Most signals emerged on the track of the previous MN trunk, indicating the reinnervation of the lower lip (Fig. 3h, video 1–4). Until 60 days, a significant increase in autofluorescence intensity was observed, suggesting the progressive collateral compensation from BN to this region. The detailed image also exhibited an obvious process of progressive collateral compensation 21 days after the operation. However, there was no signal from contralateral MN to verify whether it contributed to nerve compensation. The above findings showed that the Wallerian degeneration of the nerve appeared in the lower lip in the early stage after MN transection, and then the reinnervation process appeared.

Thus, clinical studies and animal experiments have confirmed that the BN can participate in the innervation of the sensory function of the lower lip after the alveolar nerve or MN transection, and the contralateral MN may also innervate the sensory of the lower lip midline.

### ApoD expression increased in the lower lip after mental nerve transection

**Fig. 4:**
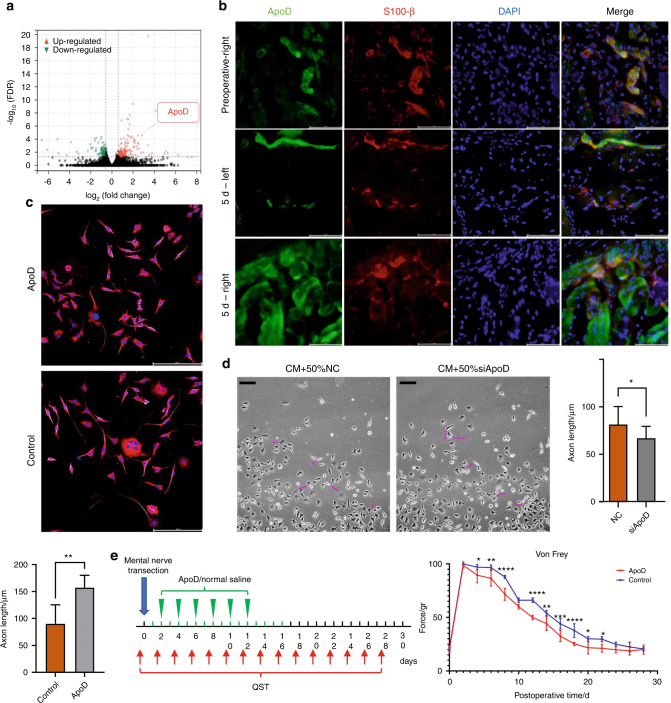
ApoD expression increased after mental nerve transection, promoting nerve growth and sensory recovery. **a** Transcriptome sequencing showed differential gene expression in the left and right lower lips of rats 5 days after mental nerve transection (*n* = 3). **b** Immunofluorescence detection of lips in rats before and 5 days after mental nerve transection. preoperative-right: Preoperative right lower lip; 5 days-left: Left lower lip 5 days after operation; 5 days-right: Right lower lip 5 days after operation (*n* = 5) (scale bar: 200 μm). **c** Immunofluorescence detection of F11 cultured with 100 ng/mL ApoD in the control group, labeled with Tuj-1. The longest 5 axons were measured with NeuronJ and analyzed quantitatively (*n* = 3) (scale bar: 200 μm). **d** Quantitative analysis of F11 axon length, cultured with 50% CM and 50% RSC96 supernatant of ApoD silencing or NC groups, respectively (*n* = 3) (scale bar: 200 μm). **e** QST analysis showed right lower lip sensation in rats with local injection of 100 ng/mL ApoD or equivalent normal saline after mental nerve transection, measured every four days (*n* = 5). *P < 0.05; **P < 0.01; ***P < 0.001; ****P < 0.000 1

Transcriptome sequencing showed that the ApoD transcription level in the lower lip of the transection side was significantly up-regulated compared with the contralateral lower lip five days after the operation (Fig. 4a). Its expression also increased significantly compared with the preoperative period and contralateral side (Fig. S2a). ApoD mainly existed around nerve fibers before transection, and it was widely found in nerves and surrounding tissues five days after transection (Fig. 4b). Compared with the clamping and ligation group, the expression of ApoD in the rats’ lower lips was higher in the transection group (Fig. S2b). In conclusion, the increased expression of ApoD suggests the occurrence and degree of nerve injury.

To explore the effect of ApoD on peripheral nerve cells, we cultured rat dorsal root ganglion (F11) cells with different concentrations of exogenous ApoD. The axon length of F11 cells increased with an increase in ApoD concentration. The axon length of F11 increased most significantly after the concentration reached 100 ng/mL (Fig. 4c, S2c). Meanwhile, the expression of Tuj-1 also increased (Fig. S2d). We silenced ApoD in RSC96 by siRNA. Compared with the NC group, F11 cells cultured with siApoD RSC96 cell supernatant exhibited shorter axons (Fig. 4d). In addition, the local injection of exogenous ApoD into the lower lip of transection rats promoted the sensory recovery of the lower lip (Fig. 4e). Thus, ApoD was involved in peripheral nerve growth and innervation.

### TGF-β regulated the expression level of ApoD in Schwann cells

**Fig. 5:**
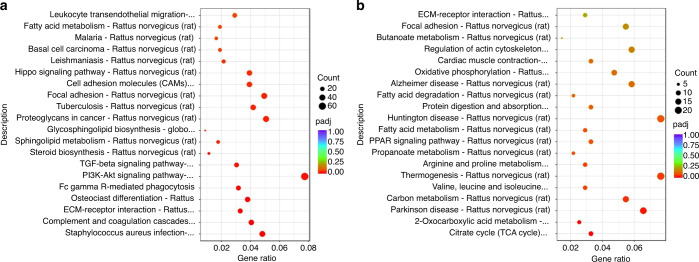
KEGG enrichment analysis after mental nerve transection. **a** KEGG enrichment analysis in rat lower lip 5 days after right mental nerve transection. **b** KEGG enrichment analysis in rat lower lip 15 days after right mental nerve transection

KEGG enrichment analysis was performed on the differential genes in the lower lip transcriptome sequencing results of right MN transection rats. Five days after the operation, there were significant differences in leukocyte trans-endothelial migration, Hippo signaling pathway, cell adhesion molecules (CAMs), focal adhesion, tuberculosis, TGF-β signaling pathway, PI3K-Akt signaling pathway, FC gamma R-mediated phagocytosis, osteoclast differentiation, ECM-receptor interaction, and complement and coagulation cascades. However, 15 days after the operation, there was differential expression of genes related to central neuropathy conditions, such as Huntington’s, Alzheimer’s, and Parkinson’s diseases. In addition, there were also differences in genes related to focal adhesion, regulation of actin cytoskeleton, oxidative phosphorylation, thermogenesis, and carbon metabolism (Fig. 5a, b).

**Fig. 6:**
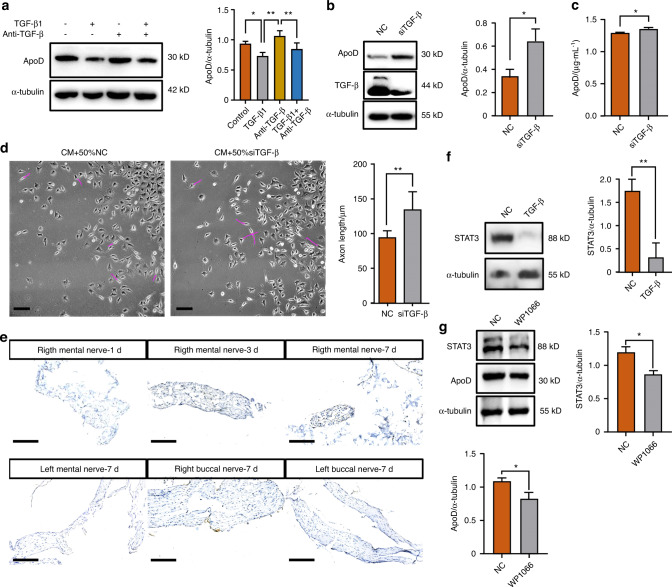
TGF-β inhibit the expression of ApoD in Schwann cells. **a** Expression of ApoD in RSC96, cultured with TGF-β1 and neutralizing antibody (*n* = 3). **b** Expression of ApoD in RSC96 stably transfected with TGF-β siRNA or control siRNA (*n* = 3). **c** ELISA analysis of ApoD secretion in RSC96 stably transfected with TGF-β siRNA or control siRNA (*n* = 3). **d** Quantitative analysis of F11 axon length, cultured with 50% CM and 50% RSC96 supernatant of TGF-β silencing or NC groups, respectively (*n* = 3) (scale bar: 200 μm). **e** Expression levels of STAT3 in the distal part of BN and MN 1, 3, and 7 days (*n* = 5, each group) after right MN transection were determined by immunohistochemical staining (scale bar: 200 μm). **f** Expression of STAT3 in RSC96, cultured with TGF-β1 (*n* = 3). **g** Expression of STAT3 and ApoD in RSC96, cultured with WP1066 (*n* = 3). *P < 0.05; **P < 0.01; ***P < 0.001; ****P < 0.000 1

TGF-β is an anti-inflammatory factor related to the level of inflammation in the early stages of nerve injury. It is involved in regulating the immune function of macrophages and glial cells. The expression of ApoD in RSC96 cultured with exogenous TGF-β decreased significantly, and adding a neutralizing antibody could block this inhibition (Fig. 6a). Silencing the expression of TGF-β in RSC96 by siRNA promoted the expression and secretion of ApoD (Fig. 6b, c, S3a). And there is no significant difference in terms of cell proliferation (Fig. S3d, e). Compared with the NC group, F11 cells cultured with siTGF-β RSC96 exhibited longer axons (Fig. 6d). Therefore, TGF-β regulated the expression and secretion of ApoD in Schwann cells, affecting peripheral nerve cell growth.

To further explore whether changes in the growth of BN and MN were induced by the phenotypic changes of Schwann cells, we collected the left and right MNs and BNs of rats 1, 3, and 7 days after the operation. Immunohistochemical results showed that Schwann cells in the distal part of BN and MN expressed STAT3, a marker related to repair-promoting phenotype, mainly concentrated in the central part of MN and the peripheral of BN 7 days after the operation (Fig. 6e). RSC96 treated with TGF-β expressed less STAT3 (Fig. 6f). In addition, ApoD expression decreased when STAT3 was inhibited by WP1066 in RSC96 (Fig. 6g).

### Zfp423, a TGF-β downstream transcription factor, inhibited the expression of ApoD in Schwann cells

**Fig. 7:**
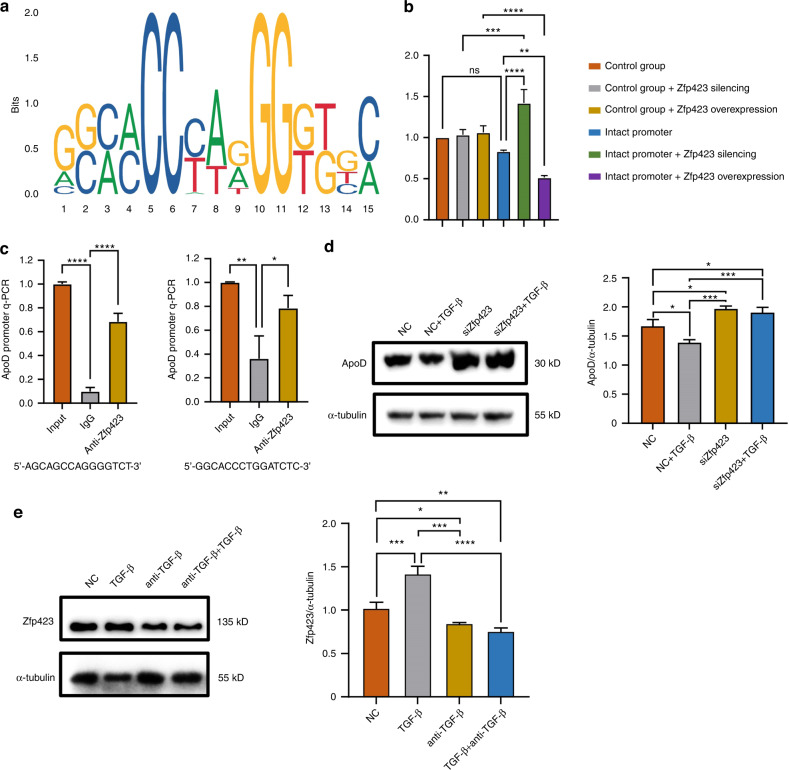
Zfp423 bind ApoD promoter to inhibit its transcription. **a** Putative Zfp423-binding motifs in ApoD promoter region. **b** Dual-luciferace results confirmed that Zfp423 silencing promoted ApoD expression and Zfp423 overexpression inhibited ApoD expression (*n* = 3). **c** ChIP-Seq analysis of binding motifs of Zfp423 (*n* = 3). **d** Expression of ApoD in RSC96 stably transfected with Zfp423 siRNA or control siRNA, cultured with TGF-β or not (*n* = 3). **e** Expression of Zfp423 in RSC96 cultured with TGF-β, anti- TGF-β or not (*n* = 3). *P < 0.05; **P < 0.01; ***P < 0.001; ****P < 0.000 1

Bioinformatics analysis showed that Zfp423 had four potential binding sites in the ApoD promoter region (Fig. 7a). To further determine whether Zfp423 could transcriptionally regulate the expression of ApoD, the activity of the ApoD promoter was tested in double luciferase report assays. Knockdown or overexpression of Zfp423 could induce or inhibit luciferase activity, respectively (Fig. 7b). Furthermore, through the ChIP-Seq experiment, we found that Zfp423 might bind to the motifs (5’-AGCAGCCAGGGGTCT-3’) and (5’-GGCACCCTGGATCTC-3’) (Fig. 7c), indicating that Zfp423 could transcriptionally regulate ApoD by binding to the ApoD promoter.

The expression of ApoD in siZfp423 RSC96 increased, and the prolongation of F11 cell axons cultured with its supernatant increased (Fig. S3b, c). The inhibition of ApoD expression by exogenous TGF-β decreased in siZfp423 RSC96 (Fig. 7d). Meanwhile, we found that TGF-β promoted the expression of Zfp423 in RSC96 (Fig. 7e), indicating that Zfp423, as a downstream factor of TGF-β, regulated the expression of ApoD.

## Discussion

Peripheral nerve injury can be triggered by mechanical injuries to neurons, adjacent nutrient vessels, or nerve fibers and is characterized by numbness, abnormal pain, etc.^21^ Nerve grafting has been proposed as the gold standard;^22^ however, sensory recovery is sometimes unsatisfactory after nerve grafting. Some studies have shown spontaneous recovery of sensation even without surgical intervention.^23^ For example, in patients with shoulder dislocation, the loss of sensation in the innervation area caused by brachial plexus injury could recover spontaneously. Patients with unrepaired finger nerve injury experienced protective sensory recovery within six months.^24,25^ A recent study also reported some cases with mandibular resection manifesting some recovery of sensation in the lower lip,^26^ suggesting that spontaneous sensory recovery after peripheral nerve injury is possible. Before this study, we found that some patients with IAN sacrifice during cancer ablation often complained about their lower lip numbness 1‒3 months after surgery. However, at the 6-month follow-up, only a few patients complained of such a disorder as they thought their lower lip sensation was almost regenerated. Therefore, we established the hypothesis that lower lip sensation could recover spontaneously without any treatment in patients with IAN sacrifice. In the present study, most patients exhibited sensory recovery of the lower lip 12 months after surgery without extra intervention. Shimizu et al.^27^ reported that seven patients with no MN reconstruction had unsatisfactory sensory recovery one year after the surgery. In that study, they adopted the Semmese-Weinstein long kit to detect tactile sense recovery at the lateral quarter of the lower lip vermilion border. However, we used the pinprick test to assess the pain sensation recovery at the lower vermilion border and skin of the lower lip. It is difficult to explain why our results do not coincide with Shimizu’s. Another study might support the present study results. Schenkel et al.^28^ included 340 facial fracture patients with confirmed IAN continuity injury requiring open reduction and internal fixation. The results showed that after 12 months, 70% of patients exhibited complete lower lip sensory recovery, and 20% experienced partial recovery, consistent with the present study results.

We also explored the potential factors that influence lower lip sensory recovery. The results showed that age, ipsilateral buccal and lingual nerve preservation, and pathological type might impact sensory recovery after IAN sacrifice. The effect of aging on sensory recovery has been confirmed as a clinical phenomenon in basic research.^29^ A similar conclusion was reached in the prognosis of patients with digital nerve repair and recovery after upper limb peripheral nerve injury repair.^30^ Aged patients often exhibit weaker regenerative and compensatory capacity due to relatively poor nutritional status and local circulation. Meanwhile, patients with ipsilateral BN and lingual nerve preservation showed a shorter recovery time and more favorable sensory recovery six months postoperatively because the ipsilateral BN can compensate for the innervation of the MN-innervated area. The trunk of the BN divides into two branches towards the upper and lower lips, forming a plexus with infraorbital and MN at the corner of the mouth.^31^ In most patients, the BN terminal branches extend to approximately one-third of the lower lip’s lateral side. BN terminal branches anastomose with the MN branches, and the ipsilateral BN can provide sensory compensation and reinnervation to the numb area based on the anastomosis, suggesting that the ipsilateral BN compensation might be part of the reason for the more favorable sensory recovery and shorter recovery time. However, communication between the MN and lingual nerve has not been elucidated. To innervate the tongue, the lingual nerve crosses the submandibular duct at the interproximal space between the mandibular first and second molars.^32^ We consider that suturing the wound brings the lingual nerve close to the buccal tissue or in direct contact with the buccal tissue, participating in the recovery of the local sensation in the subsequent repair process. In addition, the pathological type was shown to be associated with recovery time. The results showed that patients with malignant tumors tended to exhibit faster sensory recovery, which might be attributed to differences in surgical modalities between malignant and benign tumors.

Furthermore, we adopted a nerve block to test the effect of trigeminal branches on lower lip sensory recovery. The sensory loss of the ipsilateral lower lip occurs when a nerve block is adopted for the ipsilateral BN and contralateral MN, indicating that the ipsilateral BN could also grow to anastomose with the MN, contributing to the recovery of the sacrificed MN-innervated area. Furthermore, a previous study showed that the MN could cross the midline, and there is an intermediate zone for bilateral MN anastomosis.^33^ Based on this, we propose the concept of sensory nerve collateral compensation, indicating that adjacent sensory nerves could compensate for the sacrificed IAN.

To further explore the lower lip’s mechanism of sensory recovery, we established MN transection models in rats and Thy1-YFP mice. We detected the recovery of lower lip sensation by QST. The tissue-clearing technique showed that the distal cranial segment of the MN disappeared seven days after transection, and the nerve endings in the lower lip also decreased significantly. Subsequently, the BN on the same side of the injury sent out more branches and gradually entered the denervated lower lip, consistent with the results of clinical trials, providing evidence for the collateral compensation theory. By the way, among the three different types of MN injury models, the transection group exhibited the best sensory recovery compared with the clamping and ligation groups, which might be related to differences in recovery modes. It should be noted that MN and BN are two branches of the mandibular nerve given off by the trigeminal nerve and finally receives inputs from the mandibular area. Neural signals from MN and BN would realize commutation in trigeminal ganglion before central nervous system processing. These characteristics indicate that no two naturally isolated regions are innervated by the mandibular nerve. The innervated region of ipsilateral BN and MN exhibits partial overlap at the corner of the mouth. The proximity of an anatomical area is also the foundation of collateral sprouting compensation. A previous study used a partial sciatic nerve injury model to explore the collateral sprouting process. The axonal collaterals from the adjacent intact L4 spinal nerve sprouted into L3 spinal nerve degenerated nerve areas.^34^ In this model, L3 and L4 spinal nerves were two branches of the sciatic nerve trunk, reminding us to consider the collateral sprouting process of BN, consistent with our clinical results and giving us clues to follow basic experiments. Meanwhile, it is reasonable to speculate that few changes happened in the original innervated region of BN. In our clinical exploration, evaluations were focused on pain, whereas no apparent sensory variation was found in the buccal area with BN reservation and compensation. The evaluation of sensory recovery of specific regions would rely on the test of discriminative touch systems, which should be further researched. It is unclear if patients could accurately distinguish the reinnervated area from the original innervation. We confirmed that the injured MN did not contribute to the sensory recovery. One acknowledged reason is that the long-distance gap made it difficult for neurotropic factors to direct the axonal regeneration. Another reason is that nerve block assessment showed a real recovery tendency. In our rat mental nerve transection experiment, MN regeneration was not observed after transection. As mentioned in a previous study, collateral sprouting had a transcriptome profile different from axonal regeneration.

We conducted further research on rats and their cells to further investigate the potential molecular mechanism. The expression of ApoD increased significantly in the early stage after MN transection and widely existed in peripheral tissues. Compared with the clamping and ligation group, ApoD expression in the lower lip of the transection group was higher, consistent with QST results, which might be related to the inflammation scale. ApoD could induce axonal elongation of F11 cells and promote the expression of Tuj-1, which was related to the axon skeleton. Local injection of exogenous ApoD into the lower lip can also promote the sensory recovery of the lower lip. A previous study showed similar results, indicating that ApoD secreted by adipocytes could promote axonal elongation of DRG cells.^35^ ApoD could also combine with retinoic acid to enter immature nerve cells and promote neurite formation.^36^ ApoD is also involved in the stability of myelin sheath, which is closely related to the correct formation of myelin sheath structure.^37^ After ApoD is internalized by macrophages, it can regulate the phagocytosis of macrophages and improve their ability to clean up myelin fragments.^38^ This part of the experiment proves the role of ApoD in the sensory recovery of the lower lip of rats after MN resection; however, it is unknown which of the above mechanisms is involved or whether there is a new mechanism.

The TGF-β-related signaling pathway in the lower lip of the injured side changed significantly five days after the operation. Previous studies suggested that TGF-β is generally involved in anti-inflammatory effects after nerve injury.^19^ TGF-β inhibits the formation of the myelin sheath of Schwann cells and differentiates them into non-myelinated phenotypes, improving their proliferation and differentiation ability but reducing the expression of myelin-related molecules.^39^ On the other hand, TGF-β inhibition can induce human pluripotent stem cells to transform into Schwann cell precursors.^40^ The anti-inflammatory effect provides a basic environment for nerve regeneration, and the dedifferentiated Schwann cells or Schwann cell precursors are conducive to the later remyelination process. However, TGF-β in the brain was also found to inhibit neuronal morphogenesis by activating Smads and also inhibit axonal elongation of pluripotent stem cell-derived neurons and sensory neurons.^41^ Although repair Schwann cells provide signals and spatial clues to promote regeneration, they gradually lose the characteristics of regeneration support and eventually die during axon growth. STAT3 protects these cells from death and helps maintain the molecular and morphological repair phenotype that promotes axonal regeneration.^42^ Therefore, we speculate that TGF-β regulates the expression of ApoD in Schwann cells over time after MN injury by affecting the maintenance of Schwann cell repair-promoting phenotype. The bidirectional effect of TGF-β on neuronal processes may also depend on its time correlation.^39^

Zfp423 is a zinc finger transcription factor that can combine with Smads to amplify the TGF-β/ BMP signaling pathway.^43^ In the nervous system, Zfp423 is closely related to the correct morphogenesis of the cerebellum and the maturation of olfactory neurons.^44,45^ The present study showed that zfp423 could regulate the transcription of ApoD. The ChIP-Seq and luciferase report analysis showed that the binding motifs (5’-AGCAGCCAGGGGTCT-3’) and (5’-GGCACCCTGGATCTC-3’) were the functional motifs of Zfp423, mediating ApoD expression regulation. Moreover, in Zfp423-silenced RSC96, the inhibitory effect of exogenous TGF-β on ApoD expression decreased. Therefore, the TGF-β signaling pathway might regulate ApoD expression in Schwann cells through Zfp423.

## Conclusions

**Fig. 8:**
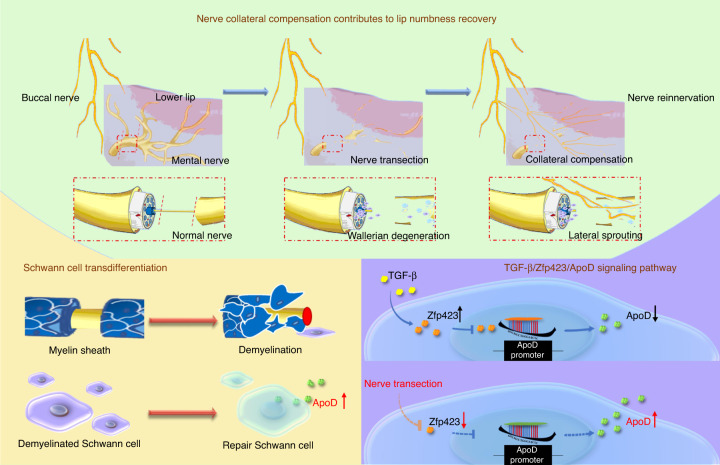
Mechanistic diagram of nerve collateral compensation contributes after nerve sacrifice. TGF-β regulate the repair-promoting phenotype of Schwann cells and regulate the transcription of ApoD in Schwann cells through Zfp423

In conclusion, we found that the lower lip sensation of patients with unilateral IAN sacrifice can recover spontaneously. The collateral compensation of the ipsilateral BN and the contralateral MN plays a key role in the recovery of sensory function. Furthermore, we found that TGF-β regulated the transcription of ApoD in Schwann cells through Zfp423, followed by regulating the morphological changes of nerve cells (Fig. 8). Therefore, regulating the expression of TGF-β at different stages may provide new ideas for studying peripheral nerve regeneration or collateral compensation.

## Materials and methods

### Prospective cohort study design and data extraction

The prospective cohort study part conformed to the STROBE checklist. The study was approved by the Medical Ethics Committee of West China Hospital of Stomatology, Sichuan University (WCHSIRB-D-2016-122). All the included patients signed informed consent forms that were used to inform them about the use of their data. The study was performed in the Department of Head and Neck Oncology, West China Hospital of Stomatology, Sichuan University. From Feb 2018 to Dec 2019, patients who underwent mandibulectomy and met the following criteria were included: (1) unilateral marginal or segmental resection of the mandible accompanied by IAN sacrifice; (2) >16 years of age. The exclusion criteria were: (1) preoperative ipsilateral IAN damage (non-cancerous); (2) removal of the ipsilateral lower lip skin during surgery; (3) contralateral IAN sacrifice or damage during surgery. Demographic and clinicopathological data were collected. Covariates included gender (female or male), age, pathological type (benign or malignant), buccal nerve (conserved or removed), lingual nerve (conserved or removed), cervical plexus (conserved or removed), radiotherapy (applied or not), chemotherapy (applied or not), types of mandibulectomy (marginal or segmental), bone graft (applied or not), lower lip incision (applied or not), and postoperative recurrence (yes or no).

### Assessment of sensory recovery and local anesthesia

Sensory recovery was assessed by a pinprick test. Patients were followed up and accepted pinprick tests by the same examiner (Li Ye) at 1-month, 3-month, 6-month, and 12-month postoperative intervals. The examiner used a probe to move away from the numb area center to all directions with the same pressure that would cause pain on the contralateral normal side of the mental skin at an interval of 1 mm. When the patient’s sense changed from numbness to pain, and the test was repeated three times with consistent results, that position was marked as a sensory boundary point. The edge of the numb area was outlined by connecting sensory boundary points. The sensory recovery proportion was calculated using the numb area at one month postoperatively as a baseline. The numb area at other follow-up intervals was divided by the baseline area to obtain sensory recovery proportion at different time intervals. Complete recovery was defined as the complete disappearance of the numb area in the lower lip. The patients were asked to contact researchers when they felt fully recovered, upon which they were arranged for another pinprick test assessment. The complete recovery time was defined as the time interval between the end of the operation and the complete recovery time point.

For some completely recovered patients who needed additional follow-ups for implant treatments, we carried out nerve block anesthesia; 0.3–0.5 mL of Articaine hydrochloride and epinephrine tartrate injection (1:100 000 epinephrine) was used to anesthetize the ipsilateral MN, contralateral MN, and ipsilateral buccal nerve to obtain an appropriate anesthetic effect. A pinprick test was performed following each anesthesia to confirm the numb area.

### Establishing three kinds of the mental nerve damage rat models

Eight-week-old male SD rats were purchased from Chengdu Dossy Experimental Animals Co., Ltd. The rats were anesthetized by intraperitoneal injection of a mixture of pentobarbital (50 mg/kg, IP). We cut the skin, subcutaneous layer, and periosteum under both mandibles until the lower edge of the mandible was exposed. Blunt dissection was performed on the anterior border of the masseter muscle and the lateral aspect of the mandible to separate the MN trunk. The right MN underwent three different treatments: transection: transection of the 4 mm MN from 1 mm outside the mental foramen; ligation: using 3-0 silk thread to tie a surgical knot 1 mm outside the mental foramen; clamping: tightening with fine tweezers at 1 mm outside the mental foramen for 15 s. The left MN was not specially treated after dissection. The wound was closed with a 3-0 silk suture.

### Local injection of exogenous ApoD

The rat model of MN resection was established and divided into two groups, with five rats in each group. The experimental group was injected with 0.05 mL of 100-ng·mL^−1^ ApoD dissolved in normal saline in the lower lip of the injured side (near the MN) 2, 4, 6, 8, 10, and 12 days after the operation, and the control group was injected with the same amount of normal saline.

### Quantitative sensory test (QST)

The rats were placed in steel wire cages, with their head out of the cage. The measuring tip of the electronic Von Frey meter was used to vertically compress the same site of the rats’ left and right lower lips, and the force was gradually applied. The behaviors of the rats, such as foot retraction, vocalization, head retraction, and avoidance, were regarded as signs, and the values of the five signs were recorded continuously for statistical analysis. Each rat was measured several times before recording to make the rat adapt to the contact of the measuring tip. We limited the maximum force to 100 gr to avoid the risk of puncturing the lower lip skin during the measurement (1 gr = 0.064 8 g).

Five rats in each group were measured before and every four days after the operation. The average of five values of each rat was taken on the same day, and two-way ANOVA was used to analyze the postoperative time and the overall difference between the groups; the Dunnett-t test was used to analyze the differences of five means between groups on the same day.

### Establishing the mental nerve transection mice model

Groups of 8‒10-week-old Thy1-YFP (B6.Cg-Tg(Thy1-YFP)16Jrs/J) mice were purchased from the Jackson Laboratory and crossed in-house. All the mice (of an approximate 20:20 mix of male and female) were housed under a regimen of 12–12 h light‒dark cycles and specific pathogen-free conditions, and all the procedures were evaluated and approved by the Ethics Committee for Animal Experimentation of the West China Hospital of Stomatology, Sichuan University (approval No.: WCHSIRB-D-2020-405). In brief, the mice were anesthetized by intraperitoneal injection with a mixture of Pentobarbital (50 mg/kg, IP). For sufficient exposure of MN, a 2-mm incision was made on the skin parallel to the anterior border of the masseter muscle. The incision continued through the skin, subcutaneous layers, and periosteum until the mandibular surface was exposed, avoiding facial artery injury. Blunt dissection was carried out above the mandible, and the MN trunk was separated. We designed a unique nerve compensation mice model by dissecting a 40-mm gap of MN on the right side to avoid this nerve’s regeneration and expose MN without cutting on the left side for the control experiment. The mice were euthanized on days 7, 14, 21, 35, and 60 after the operation. The soft tissue of the lower lip was completely resected to further experiment with the mental region. For all the groups, male and female mice were used, with no randomization method.

### Tissue clearing with the PEGASOS method

The PEGASOS method consists of multiple steps, including fixation, decolorization, delipidation, dehydration, and clearing.^34^ The whole lower lip was immersed in 4% PFA at room temperature overnight. Next, 25% N, N, N′, N′-Tetrakis (2-hydroxypropyl) ethylenediamine (Quadrol) in ddH_2_O solution was applied as the decolorizing reagent. Both the volume ratio and weight ratio are acceptable for this process. The samples were placed in 25% Quadrol for one day at a 37° shaker. Next, tert-Butanol (tB) was used for dehydration and degreasing, which contributed to better protection for YFP fluorescence. Gradient dehydration with 30%, 50%, and 70% tB supplemented with 3% Quadrol in ddH_2_O solution at a 37° shaker for four hours, six hours, and one day, respectively. For further dehydration, we adopted the tB-PEG reagent, which was composed of 75% tB + 22% poly(ethylene glycol) methacrylate (PEGMMA) + 3% Quadrol (pH = 9.0). Final dehydration with tB-PEG was carried out on a 37 °C shaker for one day, and the medium was changed once. Finally, BB-PEG medium, composed of 75% benzyl benzoate (BB), 22% PEGMMA, and 3% Quadrol, was used to achieve tissue clearing for two days. Tissue from different experiments (nerve trunk or lower lip) was imaged using a multiphoton confocal microscope (Leica) with Leica Application Suite X software. Nearly 2 mm in the z stacks and 5% overlap in the (x;y) dimensions were acquired. The motorized stage enabled the automation of the imaging procedure. All the images were then integrated using the Imaris software.

### Transcriptome sequencing

Three rats in the transection group were sacrificed 5 and 15 days after the operation, the hair of the lower lip was scraped off, and the left and right lower lips were cut and crushed, followed by storage in EP tubes with RNAlater (Beyotime). Shanghai Genechem Co., Ltd. was entrusted to conduct transcriptome sequencing, including library sequencing and biological information analysis. RNA-Seq technology was used for transcriptome sequencing, and the specific process was as follows: the total RNA of samples was extracted by a standard extraction method, and the quality of RNA was detected by agarose gel electrophoresis, nanophotometer spectrophotometry, and agilent2100bioanalyzer. mRNA with PolyA tail was enriched by oligo (DT), and the resulting mRNA was randomly interrupted by divalent cations. The first cDNA strand was synthesized with random oligonucleotides as primers, the RNA strand was degraded with RNaseH, and the second cDNA strand was synthesized with a DNA polymerase system. Purified double-stranded cDNA for end repair, added A tail and connected to the sequencing connector, screened 250–300 bp cDNA for PCR amplification. After purification, the PCR product was quantified and diluted to 1.5 ng/μL. We performed library quality inspection and conducted Illumina sequencing after passing the quality inspection.

### Cell culture

The rat Schwann cell line (RSC96) was purchased from the American Type Culture Collection (ATCC), cultured in high-glucose Dulbecco’s modified Eagle’s medium (DMEM, Servicebio), which was supplemented with 10% fetal bovine serum (GIBCO), 100-μg·mL^−1^ penicillin, and 100-μg·mL^−1^ streptomycin (Servicebio), and stored in an incubator containing 5% CO_2_. Rat embryonic dorsal root ganglion cell lines (F11) were purchased from the European Collection of Authenticated Cell Cultures (ECACC), cultured in L15 medium, supplemented with 10% fetal bovine serum (GIBCO), 100-μg·mL^−1^ penicillin, and 100-μg·mL^−1^ streptomycin (Servicebio), and stored in a CO_2_-free incubator.

### qPCR

According to the manufacturer’s instructions (Takara), total RNA was isolated from the rats’ lower lips and reverse transcribed using a cDNA synthesis kit containing gDNA remover to produce cDNA. 2X universal SYBR Green qPCR Master Mix (Servicebio) was used for qPCR on QuantStudio™3. Supplementary Table S1 lists the primers used.

### Western Blot

Protein samples extracted from the rats’ lower lips or cells were subjected to SDS-PAGE and transferred to the nitrocellulose membrane by electrophoresis for primary antibody hybridization. Mouse monoclonal Tuj-1 (1:1 000, Huabio, catalog number M0805-8, China), mouse monoclonal α-tubulin (1:5000, Signalway Antibody, catalog number 37981, China), rabbit monoclonal STAT3 (1:1 000, Huabio, catalog number ET1607-38, China), rabbit polyclonal TGF-β (1:1000, Signalway Antibody, catalog number 41494, China), mouse monoclonal ApoD (1:1 000, Santa Cruz Biotechnology, catalog number sc-373965, USA), and mouse monoclonal Zfp423/OAZ (1:1 000, Santa Cruz Biotechnology, catalog number sc-393904, USA) were used as primary antibodies. After the HRP binding of secondary antibodies (Beyotime), an ultrasensitive ECL chemiluminescence reagent (Beyotime) was used to detect protein bands on the membrane. The band intensity was quantified using ImageJ and expressed as the mean ± SD of three or five independent experiments.

### Immunofluorescence

Rat lower lips were stored in 4% paraformaldehyde. 10% sucrose solution was used for dehydration. The samples were placed in an embedding box with an OCT embedding agent and then in a −80 °C refrigerator to solidify. The samples were sliced with a layer thickness of 6 μm. 5% goat serum was added. In addition, the cells were fixed with 1% polyformaldehyde and then treated with 0.1% Triton X-100. Rabbit polyclonal anti-ApoD (1:100, Bioss, catalog number bs-1254R, China) and mouse monoclonal anti-S100-β (1:100, Bioss, catalog number bsm-10832M, China) were added as the primary antibody. Then the samples were incubated with Alexa Fluor 488 anti-rabbit or Alexa Fluor 594 anti-mouse IgG. The 4′,6′-diamidino-2-phenylindole (DAPI) was added dropwise and then sealed. Images were observed under an inverted fluorescence microscope.

### Enzyme-linked-immunosorbent serologic assay (ELISA)

The experiment was carried out according to the instructions of the rat ApoD ELISA kit (Yuanmu). OD values were measured at 450-mm wavelength.

### Gene silencing

RSC96 cells were cultured on a 6-well plate and transfected with siRNA when the cell density reached 60%. 7.5 μL of the EndoFectin™-Max (GeneCopoeia) and UltraFectin Special serum were premixed as a reducing medium for transfection (BasalMedia) of 100 μL per well and stewed for 5 min. Then, 7.5 μL of siRNA and 100 μL of UltraFectin per well were mixed with the EndoFectin-UltraFectin mixture and placed in the dark for 20 min. The medium was sucked from the 6-well plate, and 800 μL of UltraFectiUn and 200 μL of the mixture were added to each well. The transfection was observed after 4–6 h of culture in the dark. Table S1 presents the sequence of siRNA.

### ChIP-Seq

RSC96 was cross-linked in 1% formaldehyde to form a protein–DNA complex. After treatment with the lysis buffer, the DNA was broken by ultrasound. Zfp423 antibody was incubated and connected with magnetic protein A/G beads, and mouse IgG was used as an antibody negative control. Proteinase K was used to release immunoprecipitated DNA from protein–DNA complexes for qPCR identification. The ApoD promoter was identified by qPCR using the specific primers listed in Supplementary Table S1.

### Dual-luciferase reporter gene system

The promoter of ApoD was linked before the hluc sequence of the pezx-fr03 plasmid (BasalMedia). The constructs were verified by sequencing. The construct was transfected into Zfp423 silenced or overexpressed RSC96 cells (ECACC). According to the manufacturer’s instructions, firefly and Renilla luciferase activities were detected using a dual luciferase reporting analysis system.

### Axon length measurement

F11 cells were diluted and added to the center of the dried 12-well plate (about 20 μL) and cultured for 2 h to make them adhere to the wall. Then 1 mL of complete medium was added and the culture continued for 24 h. An inverted microscope was used to observe and take images of the upper, right, lower, and left growth edges. NeuronJ was used to measure and record the longest five axons in each image for analysis.

### Statistical analysis

All the statistical analyses were performed using IBM SPSS (Version 20.0.0; IBM, Armonk, NY). The association of each variable with complete recovery 12 months after surgery was analyzed by the non-parametric Mann–Whitney U-test for continuous variables and the χ^2^ test for categorical variables. Complete recovery time was analyzed by single-factor Cox regression for univariate analysis and forward stepwise Cox multivariate regression analysis for multivariate analysis, respectively. Sensory recovery proportions at 3- and 6-month postoperative intervals were analyzed as dependent variables through linear regression (stepwise). In both forward stepwise models, the entry probability was 0.10, and the removal probability was 0.15. *P* < 0.05 was considered significant for univariate and multivariate analyses.

## Supplementary information

Video1-14days

Video2-21days

Video3-60days

Video4-21days collateral compensation

Revised supplemental material
