# Supplementary material for: Promotion effect of TGF-β-Zfp423-ApoD pathway on lip sensory recovery after nerve sacrifice caused by nerve collateral compensation
Source: Int J Oral Sci. 2023 Jun 8;15:23. doi: 10.1038/s41368-023-00230-7 (PMC10247822; doi:10.1038/s41368-023-00230-7)
Supplement: Supplementary file 5 — Revised supplemental material [file 41368_2023_230_MOESM5_ESM.docx]

Supplementary information:

**Promotion effect of TGF-β-Zfp423-ApoD pathway on lip sensory recovery after nerve sacrifice caused by nerve collateral compensation**


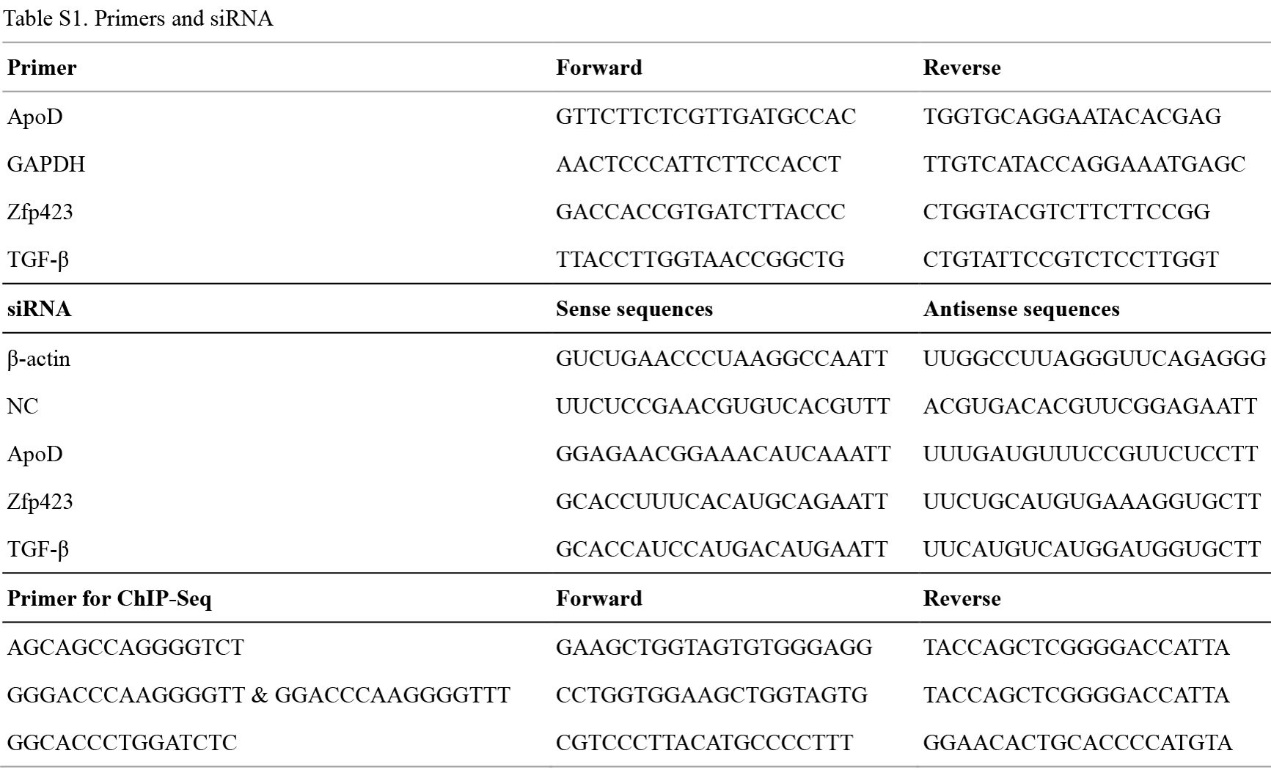


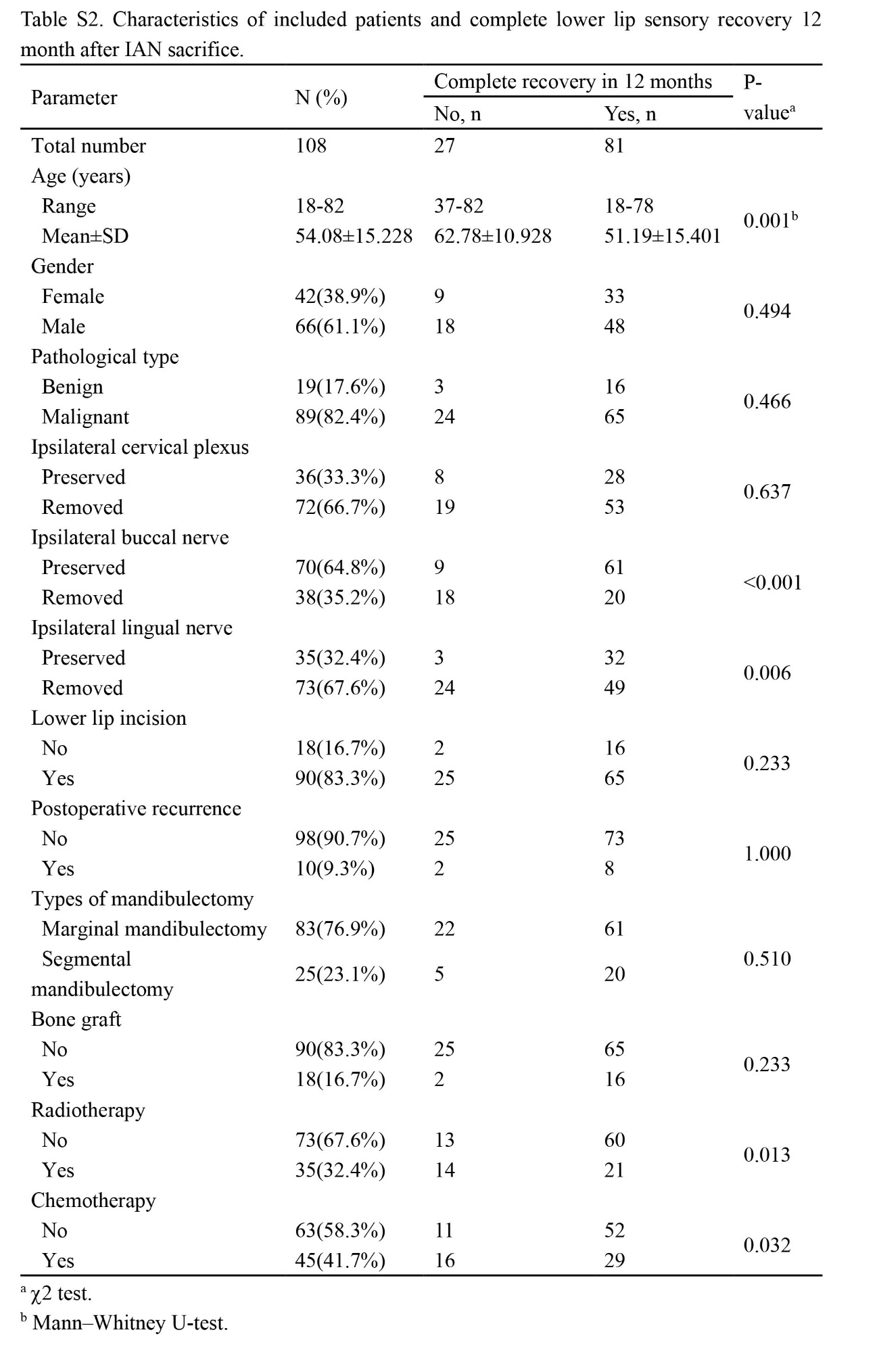

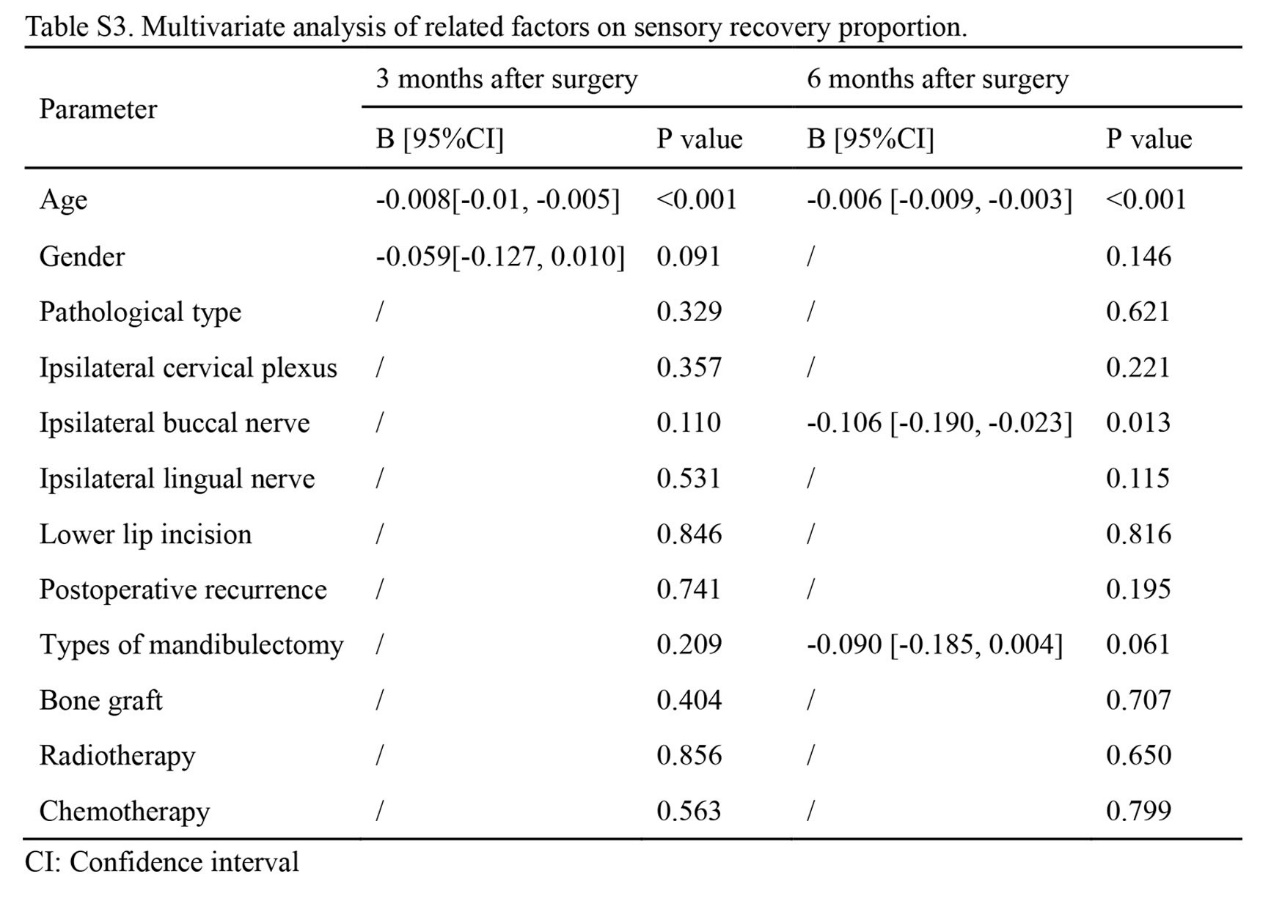


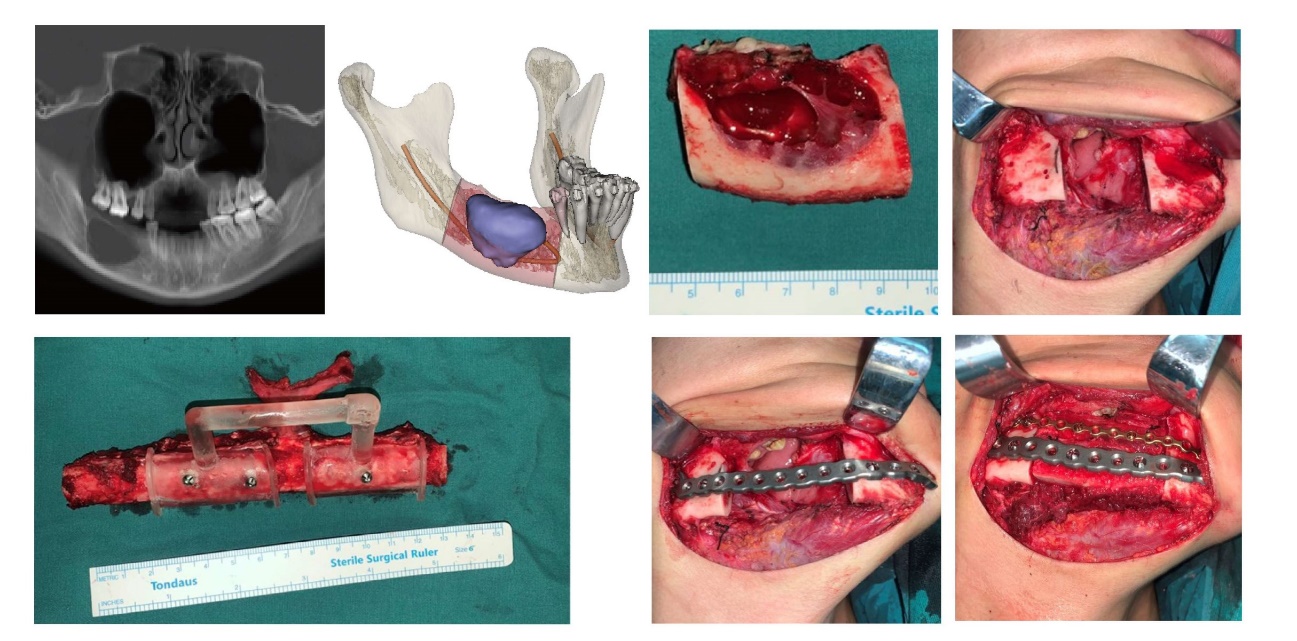


**Figure S1.** Typical mandibular segmental resection with inferior alveolar nerve sacrifice.

**
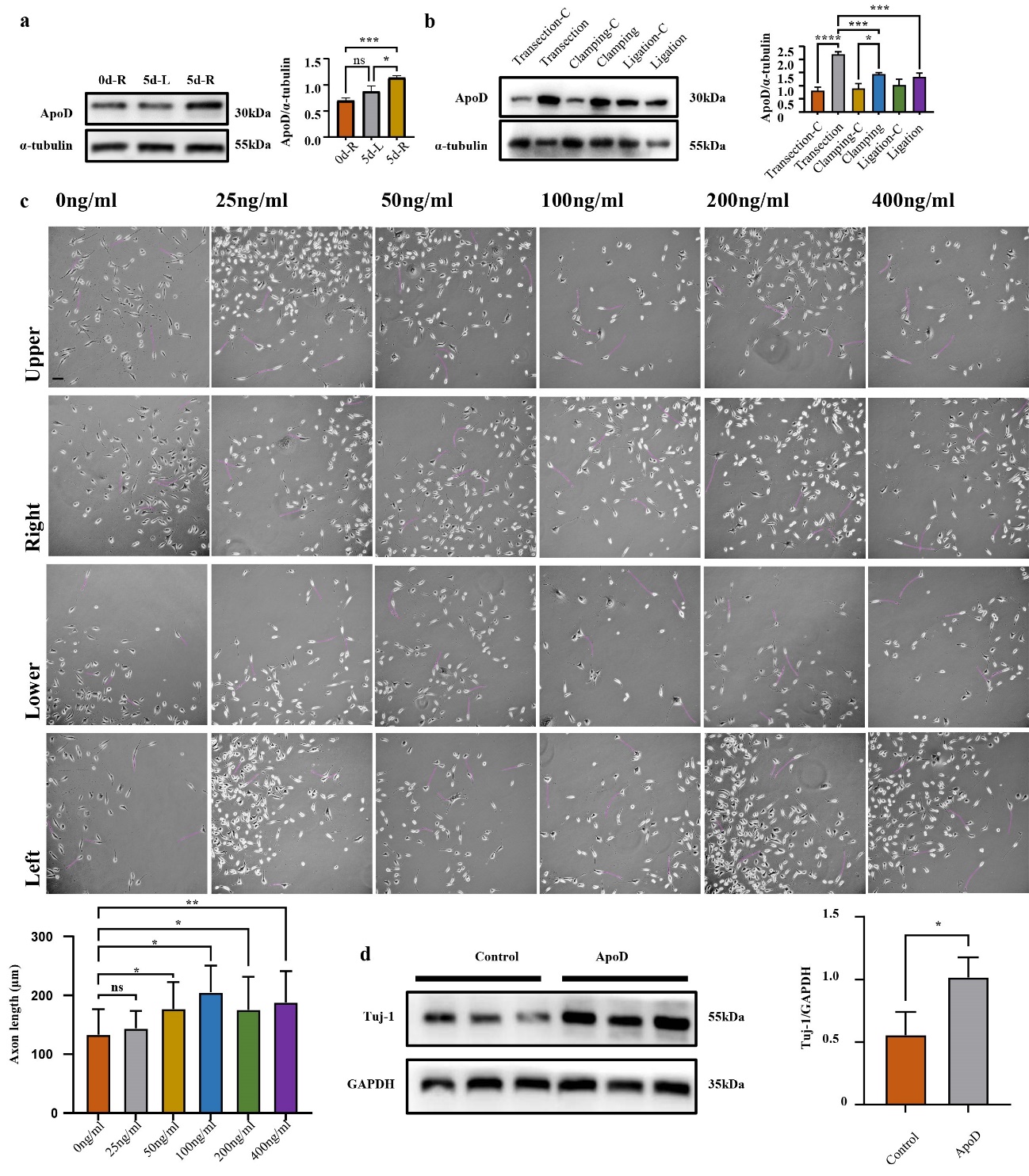
**

**Figure S2.** ApoD expression increased after mental nerve injury, promoting nerve axon growth. **a** Expression of ApoD in rat lower lips. 0d-R: Preoperative right lower lip; 5d-L: Left lower lip 5 days after right mental nerve transection; 5d-R: Right lower lip 5 days after right mental nerve transection (n=5). **b** Expression of ApoD in rat lower lips 5 days after 3 kinds of right mental nerve injury (n=5). The left mental nerve was used as a control. **c** Quantitative analysis of F11 axon length, cultured with 6 gradients of ApoD concentration (n=3). **d** Expression of Tuj-1 in F11, cultured with 100ng/ml ApoD or in control group (n=3). Note: *P < 0.05; **P < 0.01; ***P < 0.001; ****P < 0.0001. Scale bar=200 µm in panel.

**
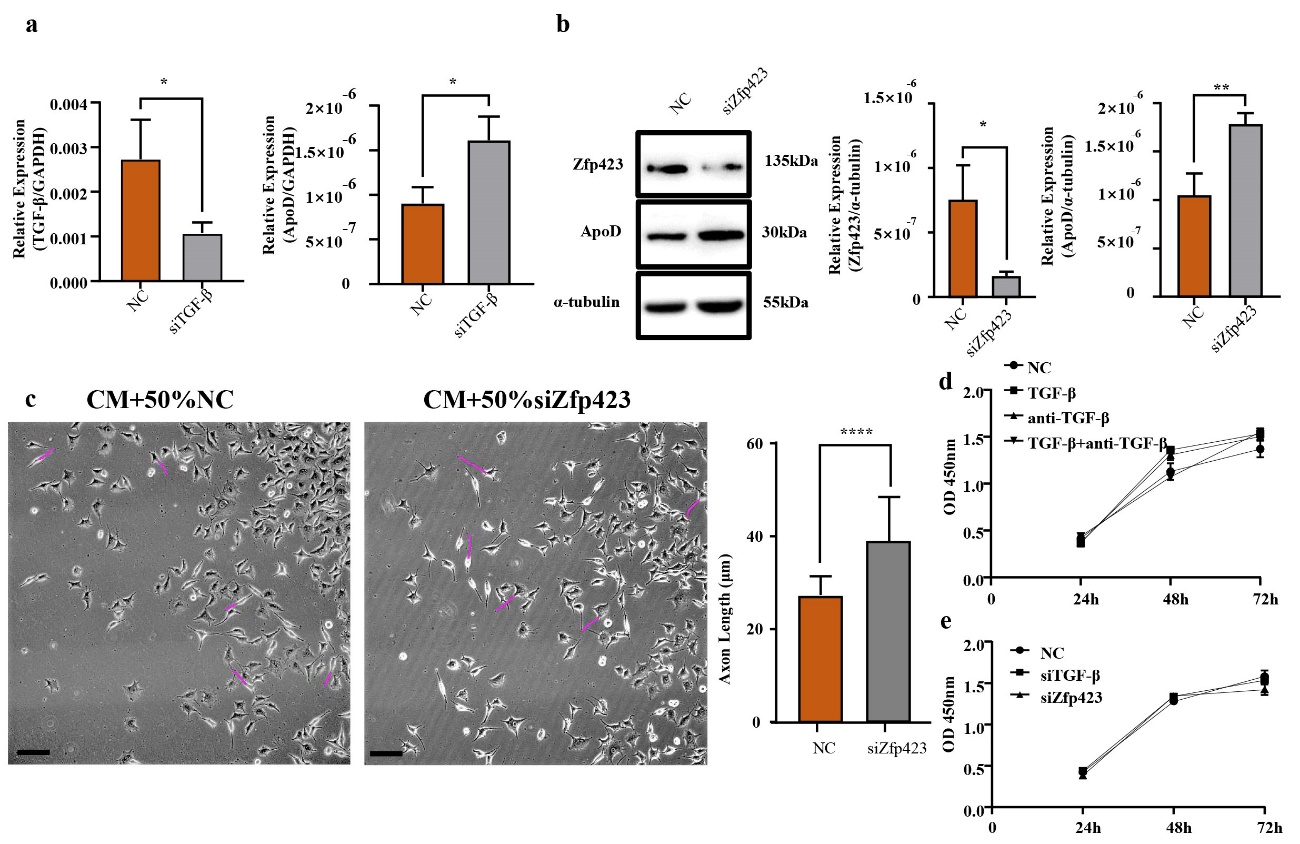
**

**Figure S3.** Zfp423 is a TGF-β Downstream transcription factor, inhibiting the expression of ApoD in Schwann cells. **a** mRNA expression of TGF-β and ApoD in RSC96 stably transfected with TGF-β siRNA or control siRNA (n=3). **b** Protein and mRNA expression of TGF-β and ApoD in RSC96 stably transfected with Zfp423 siRNA or control siRNA (n=3). **c** Quantitative analysis of F11 axon length, cultured with 50% CM and 50% RSC96 supernatant of Zfp423 silencing or NC groups, respectively (n=3). **d,e** Analysis of cell proliferation among different groups by CCK-8. Note: *P < 0.05; **P < 0.01; ***P < 0.001; ****P < 0.0001. Scale bar=200 µm in panel.
